# Stromal organization as predictive biomarker for the treatment of colon cancer with adjuvant bevacizumab; a post-hoc analysis of the AVANT trial

**DOI:** 10.1007/s13402-019-00449-9

**Published:** 2019-05-17

**Authors:** Stéphanie Zunder, Priscilla van der Wilk, Hans Gelderblom, Tim Dekker, Christoph Mancao, Anna Kiialainen, Hein Putter, Rob Tollenaar, Wilma Mesker

**Affiliations:** 1 Department of Surgery, Leiden University Medical Centre, Albinusdreef 2, 2300 RC Leiden, Netherlands; 2 Department of Medical Oncology, Leiden University Medical Centre, Leiden, Netherlands; 3 Oncology Biomarker Development, F. Hoffmann-La Roche Ltd, CH-4070 Basel, Switzerland; 4 Department of Medical Statistics, Leiden University Medical Centre, Leiden, Netherlands

**Keywords:** Colorectal neoplasms, Anti-VEGF, Tumor microenvironment, Stroma, Prediction

## Abstract

**Purpose:**

Intra-tumoral stroma has become increasingly important in understanding tumor biology, tumor progression and clinical outcome. The amount itself, quantified as the tumor-stroma ratio (TSR), has proven to be prognostic in stage I-III colon cancer. Also, alterations in stromal organization have been found to provide prognostic and predictive information in certain cancers. Here, we evaluated the predictive value of stromal organization in high-risk stage II and III colon cancer with respect to adjuvant bevacizumab and chemotherapy.

**Methods:**

In a post-hoc analysis, stromal organization was microscopically determined in hematoxylin and eosin-stained primary tumor tissue samples of 1226 patients enrolled in the AVANT trial.

**Results:**

We found that patients with tumors with a disorganized stroma showed different survival rates after the addition of bevacizumab compared to standard oxaliplatin-based chemotherapy regimens. However, overall this difference was not significant with a HR of 0.94 (95% CI 0.57–1.55; *p* = 0.80) for disease-free survival (DFS) and 1.01 (95% CI 0.51–1.99; *p* = 0.99) for overall survival (OS). Subgroup analysis, however, revealed that stromal organization combined with TSR allowed the identification of stroma-high patients with absolute cumulative survival benefits up to 15% when bevacizumab was added to oxaliplatin-based chemotherapy regimens.

**Conclusions:**

In high-risk stage II and stage III colon cancer, we found that subgroup analysis of the combined parameters stromal organization and TSR allows for the identification of patients with absolute cumulative DFS and OS benefits of up to 15%, when adding bevacizumab to the currently recommended oxaliplatin-based chemotherapy. Stromal organization itself does, however, not serve as an independent prognostic or predictive parameter.

## Introduction

Over a decade ago bevacizumab, a humanized anti-VEFG monoclonal antibody, was introduced as a new therapeutic agent for metastatic colon cancer. Since then, limited progress in systemic treatment of colon cancer has been made. Maximum utilization of existing drugs such as bevacizumab is, therefore, warranted. Currently, no validated predictive markers are available for bevacizumab and its use is only recommended in stage IV disease [1–6].

The tumor microenvironment is considered increasingly important for our understanding of tumor progression and invasion and it serves as a valuable source for potential prognostic and predictive cancer biomarkers [7–9]. Previously, we have shown that the amount of intra-tumoral stroma itself may serve as an independent prognostic biomarker for colon cancer, referred to as the tumor-stroma ratio (TSR) [10–12]. This finding has been independently validated by other groups [13, 14]. Despite its prognostic value, as of yet, evidence is lacking for TSR as a predictive biomarker for adjuvant targeted therapy [15]. However, a trend towards disease-free survival benefit was observed in patients with high stromal tumors in high-risk stage II and stage III colon cancer [16]. Currently, evidence is accumulating on the significance of intra-tumoral stroma organization in cancer. Alterations in collagen structures, such as fiber alignment, maturity, length, width and density, have shown to be prognostic in multiple cancer types [17–22]. A limited number of studies has so far focused on the predictive value of altered collagen structures. In breast cancer, Dekker et al. [20] found that patients with tumors containing highly organized collagen fibers within the intra-tumoral stroma showed a higher benefit from neoadjuvant chemotherapy than those with tumors with a disorganized stroma. This organization was microscopically evaluated and quantified using image analysis. As yet, the predictive value of this stromal organization has not been studied in colon cancer, in particular not in relation to its response to targeted therapy. Here, we evaluated whether stromal organization may serve as a biomarker to predict benefit from adjuvant targeted therapy and may improve the predictive potential of the TSR. To this end, we performed a post-hoc analysis wherein stromal organization was determined in high-risk stage II and III colon cancer patients treated with adjuvant chemotherapy plus or minus bevacizumab as part of the AVANT trial [3].

## Material and methods

### Study population

Available hematoxylin and eosin (H&E) stained tumor tissue slides from patients randomized in the AVANT trial were included in our analysis and they constituted our biomarker evaluable study population (*n =* 1226; 35.5% of AVANT intention-to-treat population). The AVANT trial is an adjuvant phase 3 (open label) randomized controlled trial that enrolled patients with high-risk stage II and stage III colon cancer. All patients underwent treatment with curative intent, including surgery (prior to randomization) followed by adjuvant chemotherapy in one of three assigned treatment arms: 5-fluorouracil/leucovorin plus oxaliplatin (FOLFOX-4) for 24 weeks followed by 24 weeks of observation, FOLFOX-4 + bevacizumab or capecitabin plus oxaliplatin (XELOX) + bevacizumab for 24 weeks followed by bevacizumab monotherapy for 24 weeks. The AVANT trial was performed in accordance with the declaration of Helsinki and the protocol was approved by local ethical review committees. For a more detailed trial design, see de Gramont et al. [3]. Additional informed consent was not required for the current study (see ethical statement below).

### Stromal organization scoring

Stromal organization was determined by digital microscopic analysis of three randomly selected intra-tumoral stroma regions in H&E stained tumor slides. The slides were first digitalized by scanning them with a digital pathology slide scanner (Philips IntelliSite Ultra Fast Scanner). Subsequently, using a 10x magnification, three intra-tumoral stroma regions were randomly selected for each slide. Image fields were required to have neoplastic cells present at all borders, as described in previous studies investigating the prognostic value of the tumor-stroma ratio in colon cancer [10, 11]. A minimum of two separate images was considered sufficient, whereas a single image was considered insufficient (for instance due to a limited amount of invasive cancer or a poor tissue quality). The selected images were loaded into Image J (Image Processing and Analysis in Java, https://imagej.nih.gov/ij/). Alongside the orientation of the stromal fibers, 10 lines were drawn on each image in order to capture the overall stromal organization of the tumor. Two observers (SZ, PW) scored the images in a blinded manner. For each image, the mean orientation of the vectors was calculated into a standard deviation. The mean standard deviation of the images was considered as a final score and measure for the stromal organization within each tumor. A low value for the standard deviation indicated a radial stromal organization (i.e., aligned stroma) and a high value indicated a broad distribution (i.e., disorganized stroma) [20].

### Statistical analysis

Statistical analyses were performed using IBM SPSS Statistics software version 23.0.

Stromal organization was converted to a categorical variable by calculating secondary cut-offs, thereby creating two stromal organization groups: 1 - aligned and 2 - disorganized. Associations between stromal organization and therapy, disease stage, age, gender, tumor-stroma ratio, CEA-level and genetic mutation status (i.e., BRAF or KRAS or DNA mismatch repair deficiency) were assessed using a univariate Cox-regression analysis. Parameters with a *p* value < 0.10 in the univariate analysis were included in multivariable analyses.

Inter-observer variability was tested using the intra-class correlation coefficient (ICC).

The Kaplan-Meier method with a log-rank test was used to analyze time-to-event endpoints. The primary endpoint was disease-free survival (DFS) and was defined as the time between randomization and recurrence, new occurrence of colon cancer or death from any cause. Event-free patients at the clinical cut-off date were censored at the last date at which they were known to be disease-free and alive. The secondary endpoint, overall survival (OS), was defined as the time from randomization to death. Patients who were alive at the clinical cut-off date were censored at the date at which they were last confirmed to be alive. Predictive analyses were performed using a Cox proportional hazards model including an interaction term between treatment arms and stromal organization. The interaction test was used to test the null hypothesis that stromal organization is not predictive for response to bevacizumab. The correlation between TSR and stromal organization was tested using Spearman’s rank coefficient. A Cox regression interaction analysis was performed between the TSR and stromal organization, with the null hypothesis that the effect of stromal organization was independent of the TSR. A *p* value < 0.05 was considered statistically significant. In order to determine an adequate sample size, a pilot analysis was performed on a random selection of 227 patients. To reach a power of 80% with an α-level of 5%, a sample size of at least 789 patients was found to be necessary.

## Results and discussion

Since the introduction of bevacizumab as a therapeutic agent for metastatic colon cancer, numerous studies have considered possibilities to expand the applicability of this agent beyond the metastatic disease group. However, thus far all studies on adjuvant bevacizumab have reported negative results, and a predictive biomarker for its efficacy has so far not been identified [1, 3, 4, 15, 16].

**Fig. 1:**
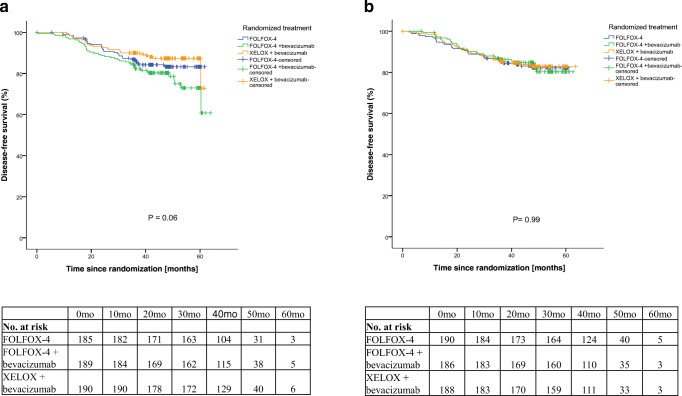
Disease-free survival: **a** Disorganized stroma, **b** Aligned stroma.  FOLFOX-4.  FOLFOX-4 + bevacizumab.  XELOX + bevacizumab

Here, we set out to investigate whether the stromal organization has predictive value for anti-VEGF therapy in high-risk stage II and III colon cancer. To this end, 1226 H&E stained tissue slides were evaluated for stromal organization with a sufficient to good level of agreement (ICC = 0.62). Subsequently, the tissues were stratified in two equal stromal organization groups based on the calculated cut-off value (Table 1). Upon predictive analysis, we found that the Kaplan-Meier-estimated 5 year DFS percentage for disorganized tumors was 57% in the FOLFOX-4 + bevacizumab group, whereas in the FOLFOX-4 monotherapy and XELOX + bevacizumab groups this was 83% and 73%, respectively (*p* = 0.06; Fig. 1a). In the aligned stroma group this difference was not observed between the three treatment groups (*p* = 0.99; Fig. 1b) Why the treatment disadvantage was only apparent in the disorganized stroma tumors treated with FOLFOX-4 + bevacizumab remains to be established. In case of a bevacizumab related effect, this would also be expected in the XELOX + bevacizumab group. The Cox proportional hazards model ruled out stromal organization as an independent predictor: hazard ratio (HR) 0.94 (95% CI 0.57–1.55; *p* = 0.80). Consequently, the interaction between stromal organization and response to therapy was not significant either (*p* = 0.25, Table 2).

**Table 1:** Patient characteristics

		Aligned stroma		Disorganized stroma		*p* value
		N	(%)	N	(%)	
Gender	Male	316	56,0	308	54,6	0.63
	Female	248	44,0	256	45,4	
Age category (years)	≤ 50	118	20,9	136	24,1	0.61
	51–64	260	46,1	264	46,8	
	65–70	125	22,2	109	19,3	
	71–80	60	10,6	54	9,6	
	> 80	1	0,2	1	0,2	
Randomized treatment	FOLFOX-4	190	33,7	185	32,8	0.95
	FOLFOX-4 + bevacizumab	186	33,0	189	33,5	
	XELOX + bevacizumab	188	33,3	190	33,7	
Disease stage	II (high-risk)	100	17,7	90	16,0	0.43
	III	464	82,3	474	84,0	
KRAS mutation	Positive	195	55,7	222	59,4	0.32
	Negative	155	44,3	152	40,6	
MMR status	MSS	431	87,1	445	89,9	0.16
	MSI	64	12,9	50	10,1	
BRAF mutation	Mutation	45	8,9	29	5,7	0.05
	Wildtype	458	91,1	476	94,3	
CEA (ng/ml)	≤ 5.0	540	97,1	549	98,2	0.23
	> 5.0	16	2,9	10	1,8	
Tumor-stroma ratio	Stroma-low	377	67,9	397	72,4	0.10
	Stroma-high	178	32,1	151	27,6	

Abbreviations: *MMR status* Mismatch Repair status, *MSI* Microsatellite instable, *MSS* Microsatellite stable, *CEA* Carcinoembryonic antigen

**Table 2:** Cox proportional hazards model with interaction between stromal organization and treatment groups

	HR (95% CI)	*p* value		HR (95% CI)	*p* value
	Disease-free survival				
Stromal organization * treatment		0.25			
Stromal organization (aligned vs. disorganized)	0.94 (0.57–1.55)	0.80			
Aligned			Disorganized		
FOLFOX-4		0.99	FOLFOX-4		0.06
FOLFOX-4 + bevacizumab	0.97 (0.59–1.60)	0.91	FOLFOX-4 + bevacizumab	1.39 (0.87–2.24)	0.17
XELOX + bevacizumab	0.97 (0.59–1.60)	0.90	XELOX + bevacizumab	0.77 (0.45–1.32)	0.34
	Overall survival				
Stromal organization * treatment		0.45			
Stromal organization (aligned vs. disorganized)	1.01 (0.51–1.99)	0.99			
Aligned			Disorganized		
FOLFOX-4		0.92	FOLFOX-4		0.08
FOLFOX-4 + bevacizumab	1.07 (0.55–2.09)	0.85	FOLFOX-4 + bevacizumab	1.52 (0.81–2.86)	0.20
XELOX + bevacizumab	0.92 (0.46–1.85)	0.82	XELOX + bevacizumab	0.70 (0.33–1.49)	0.36

**Fig. 2:**
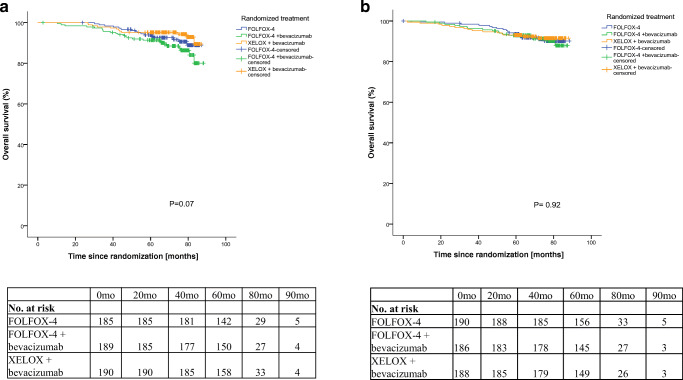
Overall survival: **a** Disorganized stroma, **b** Aligned stroma.  FOLFOX-4.  FOLFOX-4 + bevacizumab.  XELOX + bevacizumab

In the OS analysis, the previously observed difference in survival percentages between the treatment arms within the disorganized stroma group was less pronounced, with an estimated 7.5 year OS percentage of 89% in the FOLFOX-4 group, 79% in the FOLFOX-4 + bevacizumab group and 90% in the XELOX + bevacizumab group (*p* = 0.07; Fig. 2a). Within the aligned stroma group, both DFS and OS were found to be equal between the three treatment arms (*p* = 0.99; Fig. 1b, *p* = 0.92; Fig. 2b). The proportional hazards model revealed a HR 1.01 (95% CI 0.51–1.99; *p* = 0.99) for stromal organization and a non-significant interaction (*p* = 0.45, Table 2).

Regarding prognosis, no significant differences were observed for DFS (*p =* 0.99) or OS (*p =* 0.73) between patients with aligned stroma tumors versus disorganized stroma tumors, with a HR 1.03 (95% CI 0.76–1.40; *p* = 0.84) for DFS and a HR 1.06 (95% CI 0.71–1.57; *p* = 0.78) for OS in the multivariable analysis (data not shown). This observation corresponds with previously published data from Hanley et al. [21], who also ruled out collagen alignment as a significant prognosticator for cancer-specific survival in colorectal cancer. Our observation is also in agreement with a previous observation in gastric cancer [19], whereas for breast and pancreatic cancer a negative prognostic value of aligned stroma was found [22–24], suggesting that the use of stromal organization (i.e., alignment) as prognosticator may be limited to certain cancer types.

Finally, we set out to investigate whether we could improve the predictive potential of TSR through the addition of stromal organization, since we previously found a beneficial trend in distinguishing responders from non-responders to adjuvant bevacizumab using the TSR. In this study, we observed a trend towards DFS benefit in stroma-high tumors when adding bevacizumab to FOLFOX-4 chemotherapy (*p =* 0.08) [16]. For 1103 of the 1128 patients included in the current study TSR scores were available (determined as part of a previous report) [16], of which 774 (68.6%) were categorized as stroma-low and 329 (29.2%) as stroma-high. Twenty-five samples (2.2%) were not scored for TSR due to a poor histological quality.

**Fig. 3:**
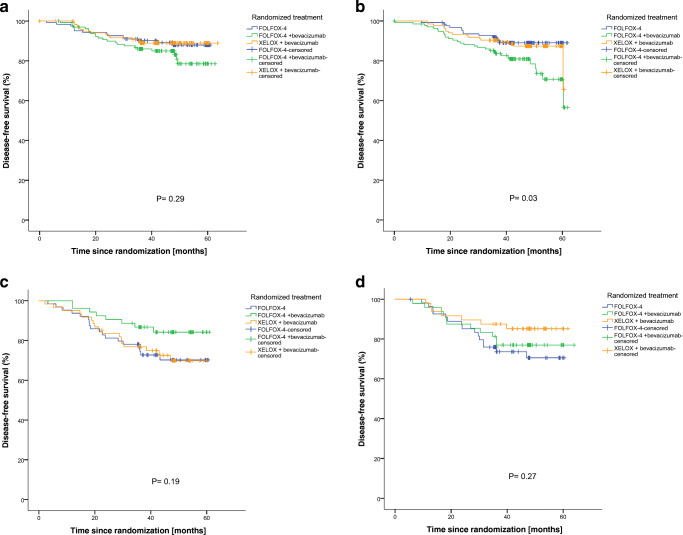
Subgroup analysis of TSR and stromal organization. Disease-free survival: **a** Stroma-low/aligned-stroma tumors, **b** Stroma-low/disorganized-stroma tumors, **c** Stroma-high/aligned-stroma tumors, **d** Stroma-high/disorganized-stroma tumors.  FOLFOX-4.  FOLFOX-4 + bevacizumab.  XELOX + bevacizumab

**Fig. 4:**
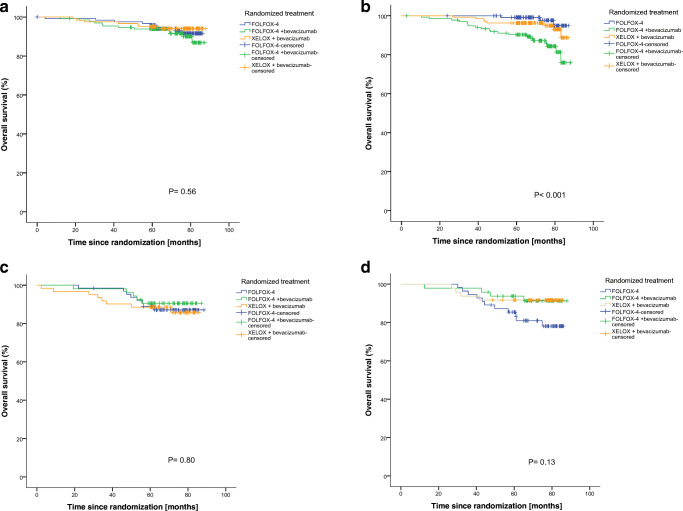
Subgroup analysis of TSR and stromal organization. Overall survival: **a** Stroma-low/aligned-stroma tumors, **b** Stroma-low/disorganized-stroma tumors, **c** Stroma-high/aligned-stroma tumors, **d** Stroma-high/disorganized-stroma tumors.  FOLFOX-4.  FOLFOX-4 + bevacizumab.  XELOX + bevacizumab

We found that patients with stroma-low tumors exhibited a significantly better DFS (*p* < 0.001) and OS (*p* = 0.02) compared to patients with stroma-high tumors (data not shown), validating the TSR as an independent prognosticator for DFS (*p* = 0.004) and OS (*p* = 0.02) in the multivariable analysis (data not shown). Spearman’s rank correlation test revealed that the TSR was not correlated to stromal organization (ρ = −0.049, *p =* 0.10). The Cox-regression interaction model ruled out an interaction between TSR and stromal organization, with a HR 0.77 (95% CI 0.43–1.39; *p =* 0.38) for DFS and a HR 0.99 (95% CI 0.44–2.21; *p* = 0.98) for OS. For subgroup analysis, we divided the data per TSR category, thereby observing noteworthy survival differences between the three treatment groups. We found that within the stroma-low tumors the stromal organization status did not affect current clinical treatment choices, since the FOLFOX-4 monotherapy group showed better, or at least equal, DFS and OS rates compared to the bevacizumab groups (Figs. 3a, b, 4a, b). However, within the stroma-high tumors the stromal organization did seem to be relevant when considering absolute cumulative survival percentages after adjuvant chemotherapy. Specifically, we found that for stroma-high/aligned stroma tumors the 5 year DFS was most favorable for the FOLFOX-4 + bevacizumab group (84%) compared to 69% in the XELOX + bevacizumab group and 71% in the FOLFOX-4 monotherapy group (Fig. 3c). Within the stroma-high/disorganized stroma group, the XELOX + bevacizumab group showed the best survival percentage (85%) compared to 77% in the FOLFOX-4 + bevacizumab group and 70% in the FOLFOX-4 monotherapy group (Fig. 3d). Despite the notable differences in survival percentages within both stroma-high subgroups, the overall log-rank tests were not significant for stroma-high/aligned stroma (*p* = 0.19) and stroma-high/disorganized stroma (*p* = 0.27) groups, respectively.

Corresponding to the DFS analysis, for the OS the overall log-rank test revealed a significant difference in the stroma-low/disorganized group (*p* < 0.001), whereas for stroma-low/aligned (*p* = 0.56), stroma-high/disorganized (*p* = 0.13) and stroma-high/aligned (*p* = 0.80) no significant differences were observed. The beneficial trend in favor of FOLFOX-4 + bevacizumab, previously found for 5 year DFS within stroma-high/aligned tumors, was now no longer observed (Fig. 4c). Within the stroma-high/disorganized tumors, however, the XELOX + bevacizumab and FOLFOX-4 + bevacizumab arms exhibited better absolute survival rates (92% and 91%, respectively) compared to FOLFOX-4 monotherapy (78%), although overall the result was not statistically significant (*p =* 0.13, (Fig. 4d). Based on these results, we conclude that implementation of stromal organization and TSR status into clinical decision making, with respect to the addition of bevacizumab, may have clinical potential, since we observed absolute survival benefits up to 15% compared to the currently recommended oxaliplatin-based chemotherapy regimens. Given the retrospective nature of our study, however, our data should be validated using other independent cohorts. One could argue that there are more advanced methods for the visualization of collagen fibers, such as second harmonic generation imaging, instead of the conventional light microscopy-based visualization method used in this study. However, since current routine practice is still mostly based on observer assessment of H&E tissue slides, the method applied in our current study could be considered as more simple to use, cost-effective and feasible. Despite being an observer dependent method, we found a good level of agreement, in concordance with Dekker et al. [20], who previously applied the current method to breast cancer analysis. Lastly, it should be noted that the current data are based on a post-hoc exploratory analysis. Although the data are derived from a well-defined prospective randomized controlled trial and the currently described study was powered for an adequate sample size, confirmatory studies are needed to definitely rule out stromal organization as a predictive parameter in colon cancer.

In conclusion, we found that stromal organization does not serve as an independent prognostic or predictive parameter in high-risk stage II and stage III colon cancer. Our work did, however, provide novel information through the subgroup analysis, although this information should be handled with care since the overall outcomes were not significant. Despite this, we found that the combination of stromal organization and TSR allowed for the identification of patients with absolute cumulative DFS and OS benefits up to 15% when adding bevacizumab to currently recommended oxaliplatin-based chemotherapy regimens.

## Abbreviations

TSR: tumor-stroma ratio
DFS: disease-free survival
OS: overall survival
FOLFOX-4: 5-fluorouracil / leucovorin / oxaliplatin
XELOX: capecitabin / oxaliplatin
